# Multisource Urban Sensing Data Fusion and Dynamic Causal Graph Modeling for Explainable Traffic State Prediction

**DOI:** 10.3390/s26144547

**Published:** 2026-07-17

**Authors:** Ran Zhu, Yingxi Wu, Xiaoya Wang, Leran Chen, Yan Zhan

**Affiliations:** 1 The Bartlett School of Architecture, University College London, London WC1E 6BT, UK; 2 Peking University, Beijing 100871, China; 3 China Agricultural University, Beijing 100083, China

**Keywords:** urban traffic congestion prediction, multisource urban sensing, artificial intelligence-driven sensing, dynamic graph neural network, reliability-aware sensor fusion

## Abstract

Urban traffic congestion prediction is an important problem in smart city sensing and intelligent traffic governance. Existing methods mostly rely on single-source traffic flow sensing data or static road topology, making it difficult to sufficiently characterize the dynamic congestion propagation process driven by multisource sensing information, such as traffic flow, vehicle trajectories, road images, public transportation, meteorological conditions, and sudden events. To address this issue, a spatiotemporal causal graph learning framework based on multisource urban sensing data is proposed for urban traffic state prediction, congestion identification, and explainable early warning. In this framework, traffic flow detector data, GPS trajectories, roadside camera data, public transportation data, weather data, and event records are first fused through a multisource urban sensing data collaborative encoding module, and the influence of low-quality or missing sensing modalities is suppressed using a reliability-aware attention mechanism. Subsequently, time-varying causal propagation relationships among road segments are adaptively learned from historical traffic states, road topology, and external disturbances through a dynamic spatiotemporal causal graph learning module. Finally, spatial diffusion and temporal evolution are jointly modeled by a causality-explanation-driven congestion prediction module, and key congestion sources, propagation paths, and inducing factors are outputs. Experimental results based on multisource traffic sensing data from the main urban area of Hangzhou show that the proposed method achieves MAE values of 3.21, 3.79, and 4.48 in 15-min, 30-min, and 60-min traffic state prediction tasks, respectively, outperforming ARIMA, XGBoost, LSTM, Transformer, STGCN, Graph WaveNet, GMAN, Multimodal Transformer, and the Causal Temporal Graph Network. In the ablation study, the complete model achieves an Accuracy of 0.914, a Precision of 0.902, a Recall of 0.889, an F1 of 0.895, and an AUC of 0.956. For congestion identification and early warning under complex scenarios, F1 values of 0.927, 0.904, and 0.893 are achieved under peak-hour, rainy-weather, and traffic-event scenarios, respectively; the corresponding AUC values reach 0.966, 0.957, and 0.948; and the false alarm rate (FAR) values are reduced to 0.061, 0.072, and 0.081. The results indicate that the proposed method can effectively improve traffic state prediction accuracy, congestion early warning reliability, and model interpretability under multisource urban sensing conditions, thereby providing an effective technical pathway for AI-driven intelligent traffic sensing.

## 1. Introduction

With the rapid growth of urbanization and vehicle ownership, traffic congestion has become an important challenge for smart city operation and intelligent transportation management [1,2]. Traffic systems exhibit complex spatiotemporal characteristics, where local disturbances may propagate through connected roads and influence regional traffic conditions [3]. Therefore, accurate congestion prediction and analysis of congestion propagation mechanisms are important for proactive traffic management and urban governance. The increasing deployment of traffic detectors, GPS devices, roadside cameras, public transportation systems, and environmental sensors has generated abundant multisource sensing data for traffic analysis [4,5]. In this study, multisource sensing data refer to heterogeneous traffic-related observations collected from different sensing platforms, including traffic flow states, vehicle trajectories, visual observations, passenger flow information, weather conditions, and traffic events. Compared with single-source traffic data, these heterogeneous observations provide complementary information about travel demand, road conditions, and external disturbances, making their integration an important research direction in intelligent transportation systems [6].

Traditional traffic prediction methods, including autoregressive integrated moving average (ARIMA), a Kalman filter, support vector regression (SVR), random forest, and XGBoost, mainly rely on historical traffic observations to estimate future states [7]. Although these methods have relatively simple structures and low computational costs, they have limited capability in modeling nonlinear traffic dynamics, long-range temporal dependencies, and external factors such as accidents, weather changes, and large-scale events. With the development of deep learning, recurrent neural networks (RNNs), long short-term memory networks (LSTMs), gated recurrent units (GRUs), Transformers, and spatiotemporal graph neural networks (STGNNs) have been widely applied to traffic prediction [8,9]. STGNNs represent urban road systems as graphs, where nodes represent road segments or intersections and edges describe spatial relationships among them. By jointly modeling temporal evolution and spatial dependencies, these methods have improved traffic prediction performance [10,11]. However, several limitations remain. First, existing methods often rely on limited traffic sensor inputs and do not fully exploit heterogeneous sensing information, such as visual data, GPS trajectories, public transportation states, and environmental factors. Second, many graph-based traffic models construct adjacency matrices according to predefined road topology or learned correlations, which may not fully describe dynamic congestion propagation patterns under changing traffic conditions. Third, existing approaches mainly focus on prediction accuracy while providing limited explanations of congestion sources, propagation paths, and influencing factors, which restricts their practical application in traffic decision-making.

Recent studies have explored multisource data fusion, knowledge-based modeling, and explainable artificial intelligence (XAI) for transportation applications [12]. Multimodal representation learning integrates complementary sensing information to improve traffic state representation [13], while explanation methods such as SHapley Additive exPlanations (SHAP) and knowledge graphs provide additional insights into prediction results [14]. However, existing approaches still lack a unified framework that jointly considers heterogeneous sensing information, time-varying congestion propagation relationships, and interpretable traffic analysis.

To address these limitations, this study proposes a spatiotemporal causal graph learning framework based on multisource urban sensing data for congestion prediction and propagation analysis. The framework first integrates traffic flow, GPS trajectories, roadside images, public transportation data, weather information, and traffic events into a unified road-level representation. A multimodal collaborative encoding mechanism is developed to learn representations from heterogeneous sensing sources, and a dynamic spatiotemporal causal graph learning module is introduced to capture time-varying congestion propagation relationships considering historical traffic states and external disturbances. Finally, graph neural networks and temporal Transformers are combined with a causal explanation mechanism to generate traffic predictions and identify potential congestion sources and propagation paths.

The main contributions of this study are summarized as follows.

1. A multisource urban traffic congestion prediction framework is proposed, which integrates traffic flow, GPS trajectories, road images, public transportation information, weather conditions, and traffic events into a unified representation for comprehensive traffic state modeling.
2. A dynamic spatiotemporal causal graph learning mechanism is developed to capture time-varying congestion propagation relationships among road segments and reduce the dependence on fixed topology structures and correlation-based graph modeling.
3. A reliability-aware multimodal encoding module is designed to adaptively fuse heterogeneous sensing information according to sensing quality and contextual consistency, improving robustness under incomplete or noisy traffic observations.
4. A causality-explanation-driven prediction mechanism is introduced to identify congestion sources, propagation paths, and influencing factors, providing interpretable support for traffic management and decision-making.

## 2. Related Work

### 2.1. Urban Traffic Congestion Prediction Based on Sensing Data

Urban traffic congestion prediction aims to estimate future road operating conditions based on historical traffic states and external environmental information [4]. It is essentially a spatiotemporal forecasting problem in which traffic dynamics are jointly influenced by temporal evolution patterns and spatial dependencies [15]. Early studies mainly relied on statistical and traditional machine learning methods [16], such as ARIMA, Kalman filters, SVR, random forest, and XGBoost, to capture temporal patterns from historical traffic data [17]. Although these approaches offer simple model structures [18], low computational costs, and a certain degree of interpretability [19], they struggle to represent the complex nonlinear spatiotemporal relationships of urban traffic systems, particularly under scenarios involving accidents, adverse weather, and large-scale events [20].

With the advancement of deep learning, models such as RNNs, LSTMs, GRUs, and Transformers have been widely adopted for traffic prediction, significantly improving forecasting accuracy through automatic spatiotemporal feature learning [21]. However, most existing studies still rely primarily on single-source traffic sensor data and insufficiently exploit multisource urban sensing information [22], including GPS trajectories, road images, public transportation data, weather conditions, and traffic event records [23]. Since traffic congestion is typically driven by the interaction of multiple factors [24], historical traffic flow alone cannot fully explain its underlying causes [25]. Consequently, multisource sensing data fusion has emerged as a key research direction in traffic prediction [26,27].

Roadside and mobile visual sensing provides complementary information that cannot be directly obtained from loop detectors or trajectory records, including vehicle accumulation, queue formation, lane occupancy, abnormal stopping, and visibility degradation. Chen et al. systematically reviewed vision-based traffic semantic understanding in intelligent transportation systems and summarized the development of vehicle detection, traffic-scene understanding, behavior recognition, and multimodal visual perception [28]. More recently, Alshehri et al. combined recurrent neural networks and Swin Transformer representations for UAV-based traffic surveillance in dynamic environments, demonstrating the potential of modern visual architectures for extracting traffic states under changing viewpoints and environmental conditions [29]. These studies confirm the value of visual sensing for traffic monitoring; however, visual features are often modeled independently from road-network dynamics or treated only as auxiliary inputs. Their reliability under rain, fog, occlusion, camera failure, and synchronization delay is also not always explicitly considered. In this study, road image representations are integrated with traffic flow, GPS trajectories, public transportation, weather, and event data through a reliability-aware multimodal fusion mechanism, enabling their contributions to be dynamically adjusted according to sensing quality and traffic context.

### 2.2. Spatiotemporal Graph Neural Networks for Urban Traffic Modeling

Given the inherent graph structure of urban road networks [30], GNNs have been widely adopted for traffic modeling by representing road segments, intersections, or monitoring stations as nodes and road connections as edges [31], thereby capturing spatial dependencies within transportation systems [32]. Building upon this framework, spatiotemporal graph neural networks, such as DCRNN, STGCN, ASTGCN, and Graph WaveNet [33], integrate temporal modeling and graph convolution to jointly learn traffic dynamics over time and spatial propagation patterns [34], achieving significant improvements in traffic prediction performance [35].

Recent studies have further explored adaptive graph construction, graph diffusion, and multi-granularity representation learning. Liu et al. proposed a multi-granularity self-guiding graph diffusion model for private-car activity prediction, in which diffusion-based graph propagation and representations at different spatial granularities are jointly modeled to capture complex mobility interactions [36]. This study demonstrates that transportation states may depend on heterogeneous spatial relationships extending beyond a single fixed graph resolution. Nevertheless, graph diffusion and multi-granularity modeling primarily improve the representation of spatial dependence and mobility correlation; they do not explicitly distinguish temporally ordered propagation effects from coincidental or common-cause correlations, nor do they directly identify event-conditioned congestion sources and propagation chains.

Despite their success, existing spatiotemporal graph models still face several limitations [37]. First, many methods rely on fixed road topologies to construct adjacency matrices [38], making it difficult to capture dynamic traffic interactions caused by changing travel demand, weather conditions, and traffic incidents [39]. Second, even adaptive graph models generally optimize predictive correlation and may assign strong edges to road segments that share recurrent temporal patterns without representing actual congestion propagation [40]. Third, most graph-based models provide limited explanations of congestion formation, propagation paths, and key intervention points [41]. To address these issues, this study introduces a dynamic spatiotemporal causal graph learning mechanism that integrates physical topology, temporal-lag dependencies, and event-conditioned disturbances to adaptively learn topology-constrained and temporally ordered propagation relationships among road segments [42], thereby improving both prediction accuracy and interpretability [43].

### 2.3. Causal Learning and Explainable AI for Smart City Transportation

With the development of intelligent transportation systems, causal learning and Explainable AI have become important directions in traffic prediction research [44]. Although deep learning models can achieve high prediction accuracy, they often lack the ability to explain the causes and propagation mechanisms of traffic congestion [45]. By distinguishing temporally ordered influence from simple association, causal learning can support the analysis of how traffic accidents, weather conditions, road construction, and signal-control changes affect future traffic states, thereby providing more reliable evidence for traffic intervention and urban governance [46].

Existing studies have applied structural causal models, Granger causality analysis, and causal graph learning to uncover relationships among traffic states, events, and environmental factors [47]. However, these approaches face challenges in modeling large-scale urban road networks, high-dimensional spatiotemporal dynamics, and heterogeneous multisource sensing data [48]. Classical causal discovery methods may also become computationally expensive when applied directly to thousands of road nodes and may depend on assumptions that are difficult to fully satisfy in real traffic environments, such as causal sufficiency, stationarity, or complete observation of confounders. Conversely, post hoc explanation methods based only on attention weights or feature gradients can identify influential inputs but do not necessarily provide physically feasible or temporally valid propagation paths.

To address these limitations, this study integrates multisource urban sensing data, dynamic graph neural networks, temporal-lag modeling, and contribution-based explanation into a unified spatiotemporal causal graph learning framework [49]. The proposed framework constrains candidate propagation relationships using road topology, temporal precedence, predictive consistency, structural sparsity, and temporal smoothness. It then combines dynamic edge weights with gradient-based contribution scores and modality-level attention weights to identify influential source roads, candidate propagation paths, and dominant external disturbances. In this manner, the framework provides explanations that are jointly supported by prediction sensitivity, graph structure, and observed traffic context rather than relying on a single post hoc importance measure.

### 2.4. Research Gap and Positioning of the Proposed Method

The above studies have advanced traffic prediction from single-variable time-series models to multimodal sensing, graph-based spatiotemporal learning, visual traffic understanding, and causal or explainable analysis. Nevertheless, four research gaps remain.

First, multisource sensing data are frequently concatenated or fused under the assumption that all modalities are continuously available and equally reliable. Existing approaches provide limited mechanisms for dynamically suppressing multiple degraded or asynchronous modalities when loop detectors, GPS trajectories, and roadside cameras simultaneously experience missing observations, noise, or synchronization latency. The proposed method addresses this gap through node- and time-specific reliability estimation and reliability-aware cross-modal attention. Second, graph-based transportation prediction methods, including adaptive graph and graph-diffusion approaches, are effective at capturing spatial dependence but mainly learn predictive correlations. They do not consistently enforce temporal precedence, event-conditioned structural variation, and sparse propagation pathways within a unified graph-learning objective. The proposed dynamic spatiotemporal causal graph combines multi-scale lag encoding, external-event modulation, physical-topology priors, sparse graph learning, and temporal-smoothness constraints to obtain time-varying propagation structures. Third, vision-based traffic sensing studies have demonstrated strong capabilities in scene understanding and traffic surveillance [28,29], but visual information is often modeled independently from road-network propagation or without explicit reliability control. The proposed framework maps roadside visual representations to road nodes and fuses them with traffic flow, trajectories, public transportation, weather, and event information in a common latent space. Fourth, many accurate traffic forecasting models provide limited operational explanations. Attention scores alone cannot verify whether an influential road segment is temporally upstream or physically connected to the target road. The proposed causality-explanation-driven prediction module combines directed dynamic edges, temporal precedence, graph propagation, gradient sensitivity, and modality contributions to produce topology-consistent candidate congestion sources, propagation paths, and inducing factors. Therefore, the main novelty of this study lies not in any single sensing modality or graph operator, but in the unified integration of reliability-aware multisource fusion, event-conditioned dynamic propagation modeling, multi-horizon forecasting, and operationally interpretable path extraction.

## 3. Materials and Methods

### 3.1. Data Collection

The data collection site was set as a typical urban road traffic network in the main urban area of Hangzhou, Zhejiang Province, China. The collection area covered urban districts with relatively dense traffic flow and diverse road functional types, including Xihu District, Gongshu District, Shangcheng District, Binjiang District, and Qiantang District, as shown in Table 1. The collection period covered one complete calendar year, from January 2024 to December 2024, including spring, summer, autumn, and winter, as well as typical traffic scenarios such as weekdays, weekends, holidays, morning and evening peak hours, off-peak periods, nighttime periods, rainfall, low visibility, road construction, and sudden traffic accidents. To ensure that multisource data could be modeled at a unified spatiotemporal scale, all data were organized using road nodes as the basic spatial units and were aggregated and aligned with a unified 5-min time window. Traffic flow data were obtained from fixed traffic flow detectors, microwave radar detectors, and geomagnetic loop sensors deployed on urban arterial roads, expressway entrances, intersection approaches, and congestion-prone road segments. These devices continuously collected road-segment-level indicators, including traffic volume, average speed, road occupancy, headway, queue length, and lane operating status. The original sampling interval was 1 min, and some high-frequency detectors could provide second-level vehicle-triggered records. To reduce the influence of short-term random fluctuations on model training, the original records were statistically aggregated using a 5-min time window during subsequent processing. Specifically, average speed, cumulative traffic flow, maximum queue length, and average occupancy within each window were calculated. GPS trajectory data were obtained from anonymized positioning records of urban taxis, ride-hailing vehicles, buses, and some commercial vehicles. The original records included desensitized vehicle identifiers, longitude, latitude, timestamps, instantaneous speed, driving direction, and positioning accuracy. The collection frequency was generally once every 5–30 s. The trajectory points were first mapped to corresponding road nodes or road segments using a map-matching method. Then, average travel time, vehicle passing frequency, route transfer intensity, interregional travel demand, and speed fluctuation features were extracted within each 5-min window.

Road image data were obtained from fixed roadside cameras and intelligent traffic cameras. The collection locations mainly included intersections, arterial road entrances, expressway ramps, areas near bus stops, and historically congestion-prone regions. The camera devices collected road operation scenes in the form of continuous video streams, and the original frame rate was generally several frames per second. Considering that direct processing of all video frames would introduce high computational cost, frame sampling was performed according to the 5-min time window, and representative road image frames were selected from each window. Vehicle density, lane occupancy ratio, queue state, abnormal stopping, road visibility, and visual congestion level were then obtained using a visual feature extraction model. Public transportation data were obtained from bus operation systems, bus-mounted positioning terminals, station passenger flow counting devices, and metro station passenger flow statistical systems. The collected information included bus arrival time, departure time, route operating speed, vehicle delay status, passenger boarding and alighting counts, transfer pressure, and metro station entry and exit passenger flow. Bus positioning data were generally uploaded every 10–30 s, while station passenger flow and metro passenger flow were summarized within 5-min or 15-min windows to characterize the influence of public travel pressure on road traffic states. Meteorological data were obtained from urban meteorological monitoring stations, road environmental sensors, and micro-meteorological monitoring devices deployed by traffic management departments. The main variables included rainfall, temperature, humidity, wind speed, air pressure, visibility, and road surface wetness. Meteorological monitoring data were generally sampled at 10-min intervals, while some road environmental sensors could provide higher-frequency road surface condition monitoring results. To ensure consistency with traffic data, meteorological data were mapped to corresponding road regions according to monitoring station locations, spatial distances to road nodes, and timestamps. Traffic event data were obtained from traffic management platforms, road event recording systems, intelligent camera-based event detection results, and manually verified records. Event types included traffic accidents, road construction, traffic control, temporary road closures, large-scale activities, abnormal parking, and sudden congestion. These data were recorded in an event-triggered manner and included event start time, end time, spatial location, event category, affected direction, impact range, and severity. The records were further expanded to corresponding time windows according to event duration. Finally, all sensing data, trajectory data, image features, public transportation states, meteorological variables, and event records were organized using unified road node identifiers, unified time windows, and unified feature formats, forming a multisource urban sensing spatiotemporal graph dataset for urban traffic congestion prediction.

### 3.2. Data Augmentation

Multisource urban traffic data can be regarded as heterogeneous spatiotemporal sequences. Traffic detectors usually collect traffic volume, average speed, and road occupancy at fixed intervals. GPS trajectories record vehicle locations at irregular sampling intervals, and their quality is often affected by sampling sparsity, positioning uncertainty, and signal interruption. Recent studies have explored trajectory completion and road-network-constrained reconstruction methods to recover missing mobility patterns and improve trajectory representation quality [50]. Roadside cameras provide high-dimensional visual observations, while weather data and event information are represented as dynamic environmental variables.

Before feature construction, a unified data quality control procedure is applied to all sensing modalities. Specifically, duplicated records, invalid timestamps, missing spatial coordinates, and physically impossible observations are removed. For traffic detector data, negative traffic volume, invalid speed values, and observations exceeding predefined physical constraints are filtered. For GPS trajectories, records with low positioning accuracy, abnormal instantaneous speed, or inconsistent movement directions are excluded before spatial aggregation. For roadside camera data, frames with transmission failures, severe occlusion, or insufficient image quality are removed before visual feature extraction. Weather and event records are also checked according to timestamp validity and spatial coverage to ensure consistency with the road-network representation.

Multisource urban traffic data can be regarded as heterogeneous spatiotemporal sequences. Traffic detectors usually collect traffic volume, average speed, and road occupancy at fixed intervals. GPS trajectories record vehicle locations at irregular sampling intervals. Roadside cameras provide high-dimensional visual observations, while weather and event information are represented as dynamic environmental variables. Therefore, all modalities are first converted into unified temporal intervals. Let the traffic state feature corresponding to the *i*-th road node at time window *t* be denoted as $x_{i}^{t}$. The overall traffic state matrix is expressed as:

(1) $$X^{t}=\left[x_{1}^{t},x_{2}^{t},\ldots ,x_{N}^{t}\right]\in R^{N\times F},$$

where *F* denotes the dimension of fused traffic features, including traffic flow, speed, occupancy, trajectory statistics, visual features, weather features, and event features. To ensure temporal consistency, a fixed time-window aggregation strategy is adopted, where continuous observations are divided into intervals with length Δt. In this study, a 5-min aggregation interval is used. For traffic detector data and GPS trajectory data, the aggregated feature is calculated as:

(2) $$x_{i}^{t}=\frac{1}{|S_{i}^{t}|}\sum_{k\in S_{i}^{t}}v_{k},$$

where $S_{i}^{t}$ denotes the set of observations associated with road node *i* within time window *t*, and $v_{k}$ represents the corresponding traffic observation value.

In terms of spatial alignment, heterogeneous sensing data are mapped into the road-network graph. Let the road network be represented as G=(V,E), where *V* denotes road nodes and *E* denotes road connectivity relationships. For a GPS trajectory point $p_{j}=\left(lat_{j},lon_{j}\right)$, nearest-neighbor mapping is adopted:

(3) $$v_{i}=\arg\min_{v\in V}d\left(p_{j},v\right),$$

where d(·) denotes the spatial distance function. This strategy is adopted because GPS trajectories are used to construct aggregated road-node-level traffic features within unified 5-min time windows rather than reconstruct complete vehicle routes or lane-level trajectories. Under this condition, nearest-neighbor mapping provides an efficient method for associating a large number of trajectory points with road units. To reduce possible mismatches on parallel roads, elevated roads, and ramps, candidate assignments are additionally screened according to positioning accuracy, vehicle heading, road direction, and a maximum distance threshold. Trajectory points that fail these consistency checks are removed before aggregation. Therefore, nearest-neighbor mapping is suitable for node-level traffic-state construction, while topology-aware Hidden Markov Model map matching is more appropriate for applications requiring complete route reconstruction or lane-level localization.

After spatial mapping, different sensing sources are converted into unified road-node-level representations.

For roadside camera data, raw videos are not directly used because of their high dimensionality and computational cost. Instead, a pretrained visual feature extraction network is used to obtain semantic traffic representations. Let the raw image input be $I_{t}$, and the visual encoder $f_{\theta }\left(\cdot \right)$ generate:

(4) $$z_{t}=f_{\theta }\left(I_{t}\right),$$

where $z_{t}$ represents visual traffic information, such as vehicle density, road occupancy, queue length, and abnormal events.

After preprocessing and quality control, the missing ratios of different sensing modalities are calculated. In the experimental datasets, traffic detector data, GPS trajectory data, roadside camera features, public transportation data, and weather/event data contain approximately 2.8%, 6.5%, 4.1%, 3.6%, and 1.9% missing observations, respectively. These missing values mainly result from temporary sensor communication failures, sparse GPS sampling, camera transmission interruptions, and incomplete external records. The missing masks are retained after completion and used as additional reliability information in the subsequent multimodal fusion module.

Since missing values and outliers inevitably exist in real-world traffic data, missing-value completion and anomaly detection are further performed. Traffic sensor failures, communication interruptions, and GPS signal occlusions can lead to incomplete observations. Directly using incomplete data may introduce incorrect traffic patterns during training. Therefore, temporal interpolation and spatial neighborhood completion are jointly adopted.

For missing values in the temporal dimension, linear interpolation is performed:

(5) $${\hat{x}}_{i}^{t}=\frac{x_{i}^{t-1}+x_{i}^{t+1}}{2}.$$

For long-term or continuous missing cases, temporal interpolation alone cannot guarantee sufficient estimation accuracy. Therefore, spatial neighboring nodes are further considered. Let N(i) denote the neighbor set of node *i*. The missing-value estimation based on spatial neighbors is expressed as:

(6) $${\hat{x}}_{i}^{t}=\frac{1}{\left|N(i)\right|}\sum_{j\in N(i)}x_{j}^{t}.$$

Here, N(i) represents the directly connected upstream and downstream road nodes defined by the directed road topology rather than all geographically close road segments. Therefore, parallel roads, elevated roads, and roads with inconsistent travel directions are not included in the neighborhood by default. This spatial completion strategy is mainly used for recovering missing observations under normal propagation conditions and is not designed to replace abnormal traffic detection. When neighboring nodes exhibit significant state differences or when accident records, construction information, abnormal stopping, queue formation, or sudden speed reductions indicate a potential localized bottleneck, the spatial average is regarded as unreliable and is not directly used as the final recovered value. In such cases, temporal observations of the target node, external event information, and graph-based spatial dependencies are jointly considered to preserve localized congestion characteristics instead of smoothing them with normal conditions from surrounding roads. Furthermore, a graph-based imputation model can be introduced to learn direction-sensitive and state-dependent propagation relationships among road nodes, allowing different neighboring roads to contribute unequally during reconstruction.

For outlier detection, both statistical rules and traffic logic constraints are adopted. Let the mean and standard deviation of a traffic feature be denoted as μ and σ, respectively. An observation is marked as a potential outlier when:

(7) $$|x_{i}^{t}-\mu |>\lambda \sigma ,$$

where λ is the anomaly detection threshold. Statistical detection alone cannot distinguish sensor errors from real traffic changes. Therefore, traffic logic constraints are further applied. For example, when the measured speed suddenly decreases to zero while no corresponding queue, accident, or abnormal event is detected from other sensing sources, the observation is considered more likely to be caused by sensor failure and is removed or corrected. Conversely, if multiple modalities consistently indicate congestion, the observation is retained as a valid traffic event.

After missing-value completion and anomaly filtering, the original missing masks, anomaly indicators, and modality quality information are preserved as auxiliary inputs rather than discarded. These indicators provide additional information for the reliability-aware fusion module, allowing the model to distinguish between reliable observations and reconstructed or uncertain features.

To improve robustness under complex traffic conditions, multiple data augmentation strategies are introduced during model training. The objective of augmentation is to simulate realistic sensing uncertainty and traffic variation without changing the fundamental characteristics of traffic dynamics.

First, random sensor masking is used to simulate temporary sensor failures. Let the original input feature matrix be *X*, and let *M* denote a random binary masking matrix. The augmented representation is: 

(8) $$\tilde{X}=M⊙X,$$

where ⊙ denotes element-wise multiplication. When $M_{ij}=0$, the corresponding observation is masked. This strategy improves robustness against incomplete sensing conditions and enables the model to learn adaptive multimodal compensation.

Second, considering that GPS trajectories may become sparse in areas with signal obstruction, such as tunnels and high-rise urban environments, trajectory sparsification is simulated. Let the original trajectory set be T and the sampled trajectory set be:

(9) $$\tilde{T}\subset T.$$

By randomly reducing trajectory density, the model learns to infer traffic states under incomplete mobility observations.

Third, weather perturbation and event variation augmentation are introduced to improve adaptability to changing environmental conditions. Let the original weather feature be $w_{t}$, and let ϵ denote a random perturbation term. The augmented weather representation is:

(10) $${\tilde{w}}_{t}=w_{t}+\epsilon ,$$

where $\epsilon ∼N(0,\sigma^{2})$ represents Gaussian noise. This operation simulates variations caused by different weather intensities, such as rainfall and visibility changes.

It should be noted that the above augmentation strategies are only applied during the training stage. The validation and testing sets retain the original data distribution without artificial masking, perturbation, or trajectory reduction. Therefore, the reported experimental results reflect the model performance under realistic sensing conditions rather than augmented samples.

Since severe congestion events are relatively rare in real traffic systems, the training data may contain class imbalance. Directly optimizing on the original distribution may cause the model to focus excessively on normal traffic states and reduce its ability to identify severe congestion. Therefore, class re-weighting is adopted during training. Let the number of samples in congestion category *c* be $n_{c}$. The corresponding class weight is defined as:

(11) $$w_{c}=\frac{1}{n_{c}}.$$

By increasing the contribution of low-frequency congestion samples, the model receives stronger supervision from abnormal traffic states and improves its capability for congestion identification under complex scenarios.

### 3.3. Proposed Method

#### 3.3.1. Overall

A spatiotemporal causal graph learning framework based on multisource urban sensing data is proposed for urban traffic congestion prediction. Given multisource road sensing features that have been temporally aligned and mapped to road nodes, each modality is first fed into its corresponding modality-specific encoder, where feature representations related to traffic flow variations, trajectory movement patterns, road visual states, public transportation pressure, weather disturbances, and event impacts are extracted separately. Let the node feature of the *m*-th modality at time *t* be denoted as $X_{t}^{m}$, and its encoded representation can be formulated as $Z_{t}^{m}=F_{m}\left(X_{t}^{m}\right)$, where $F_{m}\left(\cdot \right)$ denotes the modality-specific encoding function. Subsequently, reliability-aware fusion of different modality representations is performed by the multisource urban sensing data collaborative encoding module. According to the sensing quality, missing condition, and scenario context of each road node at the current time step, modality weights are adaptively generated, and a unified node state representation is obtained as $H_{t}=\sum_{m}\alpha_{t}^{m}Z_{t}^{m}$. This representation not only preserves variations in road speed, traffic flow, and occupancy, but also incorporates external information, including trajectory demand, visual congestion states, public transportation passenger flow, weather conditions, and event disturbances. Furthermore, the dynamic spatiotemporal causal graph learning module takes the historical node representation sequence $H_{t-L+1},\ldots ,H_{t}$, road topology priors, and external disturbance features as inputs, and learns a time-varying causal adjacency matrix $A_{t}^{c}$ to characterize the potential congestion propagation intensity among different road segments under the current traffic scenario. Unlike fixed road topology, $A_{t}^{c}$ can be dynamically adjusted according to conditions such as peak hours, rainfall, accidents, and construction. Finally, the causality-explanation-driven congestion prediction module feeds $H_{t}$ and $A_{t}^{c}$ into a graph-temporal prediction network. Key influential road-segment information is aggregated through causal graph convolution, and the long-term dependency from historical states to future traffic states is modeled using a temporal Transformer. Future speed, traffic flow, congestion level, and risk score are then predicted for 15-min, 30-min, and 60-min horizons. Meanwhile, major congestion sources, key propagation paths, and triggering factors are identified based on causal edge weights, modality attention weights, and prediction contribution scores, thereby forming a complete process of multisource sensing encoding, dynamic causal propagation modeling, and explainable congestion prediction. To provide a concise overview of the complete processing procedure, Algorithm 1 summarizes the main workflow of the proposed framework. The algorithm contains four sequential stages: multisource feature encoding, reliability-aware fusion, dynamic causal graph learning, and causality-explanation-driven prediction.
**Algorithm 1** Workflow of the Proposed Spatiotemporal Causal Graph Learning Framework**Require:** Multisource sensing data ${\left\{X^{m}\right\}}_{m=1}^{M}$, road topology prior $A^{p}$, external disturbance features *E***Ensure:** Future traffic states $\hat{Y}$, dynamic causal graph $A^{c}$, explanation results Γ 1:Align multisource sensing data into unified road-node representations. 2:Encode each modality independently:$$Z^{m}=F_{m}\left(X^{m}\right)$$ 3:Calculate reliability-aware fusion weights according to sensing quality and contextual consistency, and obtain fused node representation:$$H=\sum_{m}\alpha^{m}Z^{m}$$ 4:Extract temporal dependencies and incorporate external disturbances through temporal lag encoding and event-conditioned modulation. 5:Construct dynamic causal graph by estimating time-varying edge weights:$$A_{t}^{c}=f\left(H_{t},A^{p},E\right)$$ 6:Optimize causal graph learning with lag consistency, sparsity, and temporal smoothness constraints. 7:Perform causal graph propagation and temporal dependency modeling using GNN and Transformer. 8:Generate multi-horizon traffic predictions:$$\hat{Y}=\left\{Y_{15},Y_{30},Y_{60}\right\}$$ 9:Calculate causal contribution scores to identify congestion sources, propagation paths, and influencing factors.10:**return** $\hat{Y}$, $A^{c}$, Γ

#### 3.3.2. Multisource Urban Sensing Data Collaborative Encoding Module

The multisource urban sensing data collaborative encoding module takes multimodal sensing features that have been temporally aligned, spatially mapped, and organized at the road-node level as inputs. Let the urban road network contain *N* nodes, let the historical window length be *L*, and let the input of the *m*-th modality be denoted as $X^{m}\in R^{N\times L\times d_{m}}$, where $d_{m}$ is the original feature dimension of that modality. Because traffic flow, GPS trajectories, road images, public transportation, weather, and event data differ in physical meaning, sampling frequency, and noise characteristics, each modality is processed by an independent encoder and then projected into a shared latent space.

For continuous time-series modalities, including traffic flow, speed, occupancy, trajectory-derived travel time, and public transportation operating states, a one-dimensional temporal convolution followed by a gated recurrent unit is used. The temporal convolution captures local variations, while the recurrent unit models dependencies across time steps. The encoded representation is written as

(12) $$Z^{m}=F_{m}\left(X^{m};\Theta_{m}\right),$$

where $F_{m}\left(\cdot \right)$ and $\Theta_{m}$ denote the encoder and its parameters for modality *m*. For roadside-camera features, a linear projection layer and a temporal attention layer are used to compress frame-level representations and align them with node-level traffic sequences. Weather and event variables are first embedded and then broadcast to the road nodes within their corresponding spatial coverage. After encoding, all modalities are represented as $Z^{m}\in R^{N\times L\times d}$, where *d* is the common latent dimension.

**Figure 1:**
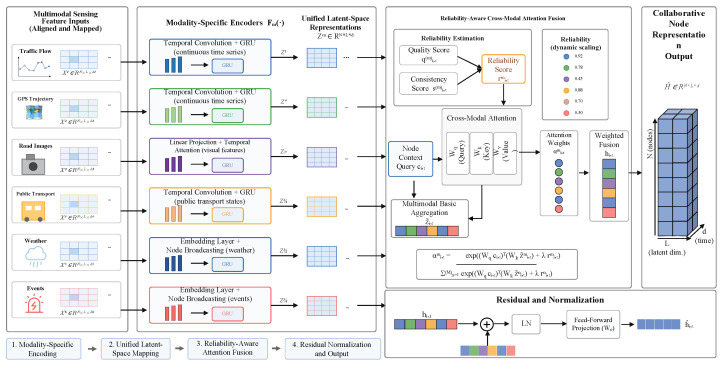
The multisource urban sensing data collaborative encoding module generates road-node-level representations through modality-specific encoding, shared latent-space mapping, reliability-aware cross-modal attention, and residual normalization.

As shown in Figure 1, the reliability of each modality is estimated separately for every node and time step. For the representation $z_{i,t}^{m}$ of modality *m* at node $v_{i}$ and time *t*, the reliability estimator considers the observation quality, missing-data status, temporal consistency, and spatial consistency. Let $a_{i,t}^{m}\in \left\{0,1\right\}$ denote the availability mask, where $a_{i,t}^{m}=0$ indicates that the observation is missing. The quality feature is defined as

(13) $$q_{i,t}^{m}=a_{i,t}^{m}\left(1-\nu_{i,t}^{m}\right),$$

where $\nu_{i,t}^{m}\in \left[0,1\right]$ is the normalized noise or uncertainty level estimated from modality-specific indicators, such as detector error rate, GPS positioning accuracy, image quality, or packet-loss ratio. Thus, unavailable or low-quality observations obtain smaller quality values.

The consistency score measures whether the current representation agrees with its temporal and spatial context. It is calculated as

(14) $$s_{i,t}^{m}=\frac{1}{2}\left[\exp\left(-\frac{{∥z_{i,t}^{m}-z_{i,t-1}^{m}∥}_{2}^{2}}{\tau_{t}}\right)+\exp\left(-\frac{{∥z_{i,t}^{m}-{\bar{z}}_{N(i),t}^{m}∥}_{2}^{2}}{\tau_{s}}\right)\right],$$

where $\tau_{t}$ and $\tau_{s}$ are scaling parameters and

(15) $${\bar{z}}_{N(i),t}^{m}=\frac{1}{\left|N(i)\right|}\sum_{j\in N(i)}z_{j,t}^{m}$$

is the mean representation of the topologically connected neighboring nodes. The first term measures temporal continuity, while the second term measures spatial consistency. A large deviation from both the preceding state and neighboring nodes results in a lower consistency score.

The reliability coefficient is then computed as

(16) $$r_{i,t}^{m}=a_{i,t}^{m}\;\sigma \left(W_{r}\left[z_{i,t}^{m};q_{i,t}^{m};s_{i,t}^{m}\right]+b_{r}\right),$$

where [·;·] denotes feature concatenation and σ(·) is the sigmoid function. The availability mask ensures that a missing modality receives a reliability value of zero, while the learned transformation allows the model to combine modality content, observation quality, and contextual consistency. The reliability coefficient is incorporated into the cross-modal attention logits. For modality *m*, the unnormalized attention score is defined as

(17) $$e_{i,t}^{m}={\left(W_{q}c_{i,t}\right)}^{⊤}\left(W_{k}z_{i,t}^{m}\right)+\lambda r_{i,t}^{m},$$

where $c_{i,t}$ is the query vector obtained from the multimodal context and λ controls the influence of reliability. The normalized modality weight is

(18) $$\alpha_{i,t}^{m}=\frac{a_{i,t}^{m}\exp\left(e_{i,t}^{m}\right)}{\sum_{u=1}^{M}a_{i,t}^{u}\exp\left(e_{i,t}^{u}\right)+\varepsilon },$$

where ε is a small constant used for numerical stability. The weights satisfy $\sum_{m=1}^{M}\alpha_{i,t}^{m}=1$ when at least one modality is available. A modality with missing observations, poor sensing quality, or weak temporal-spatial consistency therefore receives a smaller fusion weight, while the weights of the remaining available modalities increase after normalization. The fused node representation is calculated as

(19) $$h_{i,t}=\sum_{m=1}^{M}\alpha_{i,t}^{m}W_{v}z_{i,t}^{m}.$$

To retain information from the basic multimodal aggregation and stabilize training, the fused representation is further processed using a residual connection, layer normalization, and feed-forward mapping:

(20) $${\hat{h}}_{i,t}=\mathrm{LN}\left(h_{i,t}+W_{o}{\bar{z}}_{i,t}\right),$$

where ${\bar{z}}_{i,t}$ denotes the basic aggregation of the available modality representations.

This design allows modality contributions to vary across nodes and time steps according to the observed data quality and traffic context. For example, rain, fog, or occlusion may reduce the quality and consistency scores of roadside-camera features, thereby lowering their attention weights. Similarly, sparse GPS observations or missing public transportation records receive smaller reliability values, while available traffic detectors, event records, and neighboring-node information receive larger normalized weights. The resulting representation preserves traffic-state, travel-demand, visual, weather, and event information in a common latent space and is used as the input to the subsequent dynamic graph learning module.

#### 3.3.3. Dynamic Spatiotemporal Causal Graph Learning Module

The dynamic spatiotemporal causal graph learning module is used to learn time-varying congestion propagation structures based on multisource node representations.

**Figure 2:**
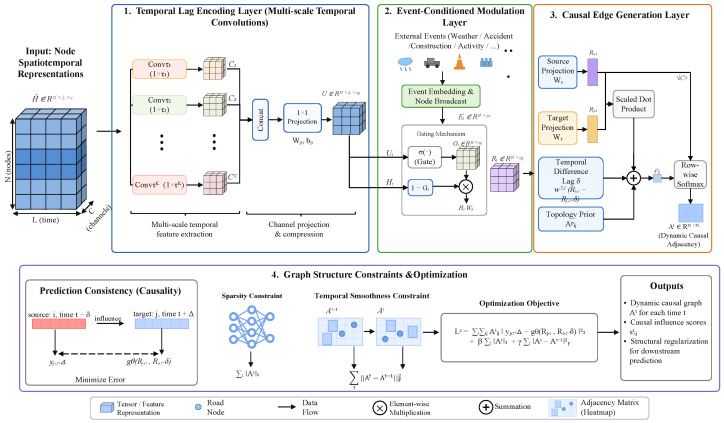
The dynamic spatiotemporal causal graph learning module adaptively learns dynamic congestion propagation relationships under different traffic scenarios through multi-scale temporal lag encoding, event-conditioned modulation, causal edge generation, and graph structure constraints.

As shown in Figure 2, let the node spatiotemporal representation output by the previous module be denoted as $H\in R^{N\times L\times C}$, where *N* is the number of road nodes, *L* is the historical time window length, and *C* is the input channel dimension. This module is designed as a multilayer structure consisting of a temporal lag encoding layer, an event-conditioned modulation layer, a causal edge generation layer, and a graph structure constraint layer. The temporal lag encoding layer contains *K* parallel temporal convolution branches. The *k*-th branch has a convolution kernel size of $1\times \tau_{k}$, an input channel number of *C*, and an output channel number of $C_{k}$ and is used to capture sequential influences of road state changes at different temporal scales. After all branches are concatenated, a 1×1 projection layer is used to compress the representation into $C_{g}$ channels, resulting in the node lag representation $U\in R^{N\times L\times C_{g}}$. This process can be expressed as:

(21) $$U=\phi \left(W_{p}*\mathrm{Concat}\left({\mathrm{Conv}}_{\tau_{1}}\left(H\right),\ldots ,{\mathrm{Conv}}_{\tau_{K}}\left(H\right)\right)+b_{p}\right),$$

where ${\mathrm{Conv}}_{\tau_{k}}\left(\cdot \right)$ denotes temporal convolution, $W_{p}\in R^{1\times 1\times \sum_{k}C_{k}\times C_{g}}$ denotes the channel projection parameter, and ϕ(·) denotes the nonlinear function. Subsequently, weather, accidents, construction, activities, and other external disturbances are represented by the event-conditioned modulation layer as $E_{t}\in R^{N\times C_{e}}$, and the causal expression of nodes is adjusted through a gating mechanism:

(22) $$G_{t}=\sigma \left(E_{t}W_{e}+U_{t}W_{u}+b_{g}\right),\;R_{t}=G_{t}⊙U_{t}+\left(1-G_{t}\right)⊙H_{t}W_{h}.$$

Here, $W_{e}\in R^{C_{e}\times C_{g}}$, $W_{u}\in R^{C_{g}\times C_{g}}$, and $W_{h}\in R^{C\times C_{g}}$. This design enables external disturbances to directly affect the causal edge generation process rather than being passively input as ordinary auxiliary features. For any pair of road nodes $v_{i}$ and $v_{j}$, causal influence intensity is jointly calculated through source node projection, target node projection, and directional lag difference:

(23) $$s_{ij}^{t}=\frac{\left(R_{i,t}W_{s}\right){\left(R_{j,t}W_{r}\right)}^{⊤}}{\sqrt{C_{g}}}+w_{d}^{⊤}\left(R_{j,t}-R_{i,t-\delta }\right)+\eta A_{ij}^{p},$$

where $W_{s},W_{r}\in R^{C_{g}\times C_{a}}$, $C_{a}$ denotes the edge attention channel number, δ denotes a learnable or candidate time lag, $A_{ij}^{p}$ denotes the physical topology prior, and η denotes the topology adjustment coefficient. The dynamic causal adjacency matrix is obtained through a normalization function:

(24) $$A_{ij}^{t}=\frac{\exp(s_{ij}^{t})}{\sum_{q=1}^{N}\exp\left(s_{iq}^{t}\right)}.$$

During training, the causal relationship between nodes $v_{i}$ and $v_{j}$ is estimated in each mini-batch from the current event-conditioned node representations. Specifically, the source and target projection parameters $W_{s}$ and $W_{r}$, the directional lag parameter $w_{d}$, the event-modulation parameters, and the topology coefficient are optimized jointly with the downstream prediction network. At each forward pass, the model first computes the edge score $s_{ij}^{t}$ and then normalizes all candidate outgoing relationships of node $v_{i}$ to obtain $A_{ij}^{t}$. Therefore, the adjacency matrix is not a fixed trainable parameter; it is dynamically regenerated from the current traffic states, temporal-lag information, road topology, and external disturbances for every time step and training sample.

The edge weights are updated through backpropagation from both the causal graph loss and the final traffic-prediction loss. If a candidate edge helps reduce the future-state prediction error of its target node, the gradient increases the compatibility between the corresponding source and target representations and may increase its normalized weight. Conversely, if the edge provides little lagged predictive information or produces a large target-state residual, its score is reduced during parameter updating. Because the softmax normalization couples the candidate edges associated with the same source node, increasing the weight of a more informative relationship simultaneously decreases the relative weights of less informative alternatives. The event-conditioned gate also allows the same node pair to receive different edge weights under peak-hour, rainfall, accident, or normal operating conditions.

To make the learned graph structure closer to causal propagation rather than static correlation, directional lag consistency constraints and sparse smoothness constraints are introduced. If a causal influence exists from node *i* to node *j*, then the past state changes of the source node should reduce the uncertainty of the future state of the target node. Therefore, the following optimization objective is formulated:

(25) $$L_{c}=\sum_{t}\sum_{i,j}A_{ij}^{t}{\left|y_{j,t+\Delta }-g_{\theta }\left(R_{j,t},R_{i,t-\delta }\right)\right|}_{2}^{2}+\beta \sum_{t}{\left|A^{t}\right|}_{1}+\gamma \sum_{t}{\left|A^{t}-A^{t-1}\right|}_{F}^{2}.$$

The complete network is trained end-to-end using mini-batch gradient descent. For each batch, the dynamic graph is first generated from the current node representations, after which graph propagation and multi-horizon prediction are performed. The prediction loss and $L_{c}$ are then jointly differentiated with respect to the graph-generation and prediction parameters. The sparsity term removes weak and redundant edges, while the temporal smoothness term limits unnecessary changes between adjacent graph snapshots. Consequently, causal relationships are updated iteratively according to their lagged predictive contribution, structural necessity, and temporal stability rather than being estimated once before model training. At inference time, the learned parameters are fixed, but a new dynamic adjacency matrix is still generated from the observed traffic and event conditions at each prediction window.

The proposed objective mitigates overfitting to spurious correlations through the complementary effects of lagged predictive consistency, structural sparsity, and temporal smoothness. Specifically, the first term requires the historical state of a candidate source node $R_{i,t-\delta }$ to provide additional information for predicting the future state of the target node $y_{j,t+\Delta }$ after the current target representation $R_{j,t}$ has been considered; therefore, purely synchronous or repetitive correlations that do not improve future prediction tend to produce larger residuals and receive lower edge weights. The $\ell_{1}$ sparsity penalty further suppresses weak and redundant connections caused by shared peak-hour, weather, or regional-demand patterns, while the temporal smoothness term prevents isolated sensor noise and accidental short-term co-fluctuations from generating abrupt graph changes. Together with the physical-topology prior and directional time-lag modeling, these constraints encourage the learned graph to retain parsimonious, temporally ordered, and stable predictive propagation relationships. Nevertheless, because unobserved confounders cannot be completely excluded, the learned edges are interpreted as topology-constrained causal candidates rather than fully identified interventional causal effects.

The first term constrains causal edges to contribute to the explanation of the future state of the target node; the second term avoids meaningless fully connected propagation, and the third term ensures smooth variation in graph structures between adjacent time steps. Its rationality can be explained from the perspective of empirical risk minimization. When $A_{ij}^{t}$ is large, the optimization process forces $R_{i,t-\delta }$ to provide a stronger predictive contribution to $y_{j,t+\Delta }$. If an edge only reflects synchronous correlation without providing lagged predictive information, its corresponding residual becomes large, and the edge weight is reduced under the sparsity penalty. Therefore, the learned $A^{t}$ tends to preserve road influence paths with temporal precedence and predictive explanatory capability. When applied to the proposed task, this module can overcome the limitations of fixed road topology and adaptively adjust congestion propagation structures under different scenarios, such as morning and evening peaks, rainfall, accidents, and construction. Consequently, the model can identify not only which roads are adjacent, but also which roads drive congestion diffusion under current conditions, thereby improving the reliability of long-horizon prediction, sudden-event prediction, and congestion propagation explanation.

#### 3.3.4. Causality-Explanation-Driven Congestion Prediction Module

The causality-explanation-driven congestion prediction module is used to transform the dynamic spatiotemporal causal graph into predictable and interpretable future traffic state outputs.

**Figure 3:**
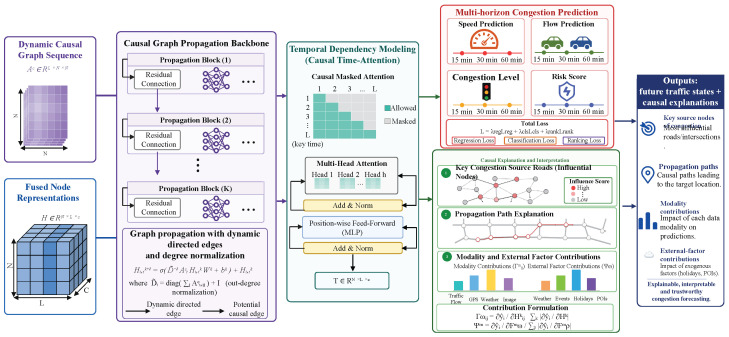
The causality-explanation-driven congestion prediction module performs future traffic state prediction and identifies congestion sources, propagation paths, and influencing factors through dynamic causal graph propagation, causal temporal attention, multi-horizon prediction, and contribution explanation.

As shown in Figure 3, let the dynamic causal graph sequence obtained from the previous module be denoted as $A^{c}\in R^{L\times N\times N}$, and the multisource fused node representation be denoted as $H\in R^{N\times L\times C}$. This module is designed as an end-to-end network structure consisting of a causal graph propagation layer, a temporal dependency modeling layer, a multi-task prediction layer, and a causal contribution explanation layer.

The causal structure used in this module follows a temporally ordered structural-prediction formulation. For a target node $v_{j}$, its future traffic state is modeled as a function of its current state, the lagged representations of candidate upstream nodes, and observed external disturbances, including weather and traffic events. Accordingly, the dynamic edge $A_{ij,t}^{c}$ represents the conditional predictive contribution of $R_{i,t-\delta }$ to the future state of $v_{j}$ after the current representation of the target node and the observed contextual variables have been considered. Rather than applying a separate constraint-based conditional independence algorithm, such as PC or FCI, the proposed framework evaluates this conditional contribution through the lagged prediction-consistency objective in the graph-learning module. Candidate relationships that do not provide additional future-state information beyond the target node’s own history are assigned lower edge weights under the prediction, sparsity, and temporal-smoothness constraints.

The causal graph propagation layer adopts *B* stacked dynamic graph propagation blocks. Each block has an input width of *N* nodes, a height of *L* historical time steps, an input channel number of $C_{b}$, and an output channel number of $C_{b+1}$. In the *b*-th layer, node features are first transformed into query states through channel mapping, and then directed propagation is performed along the dynamic causal graph. This process is expressed as:

(26) $$P_{t}^{\left(b+1\right)}=\rho \left(D_{t}^{-\frac{1}{2}}A_{t}^{c}D_{t}^{-\frac{1}{2}}P_{t}^{\left(b\right)}W_{b}+P_{t}^{\left(b\right)}W_{r}^{b}+b_{b}\right),\;$$

where $P_{t}^{\left(b\right)}\in R^{N\times C_{b}}$ denotes the node representation of the *b*-th layer at time *t*, $D_{t}$ denotes the degree matrix of the causal graph, and $W_{b}\in R^{C_{b}\times C_{b+1}}$ and $W_{r}^{b}\in R^{C_{b}\times C_{b+1}}$ denote the causal propagation mapping parameter and residual mapping parameter, respectively. ρ(·) denotes the activation function. This structure ensures that the target road node receives not only its own historical state, but also information from upstream congestion sources, adjacent detour roads, and event-affected regions according to the direction of causal edges.

After spatial causal propagation is completed, the representation of each node over the *L* historical time steps is fed into the temporal dependency modeling layer. This layer adopts *S* layers of causal temporal attention structures, each of which contains multi-head temporal attention and feed-forward mapping. The input tensor size is $N\times L\times C_{g}$, and the output size is $N\times L\times C_{o}$. To avoid future information leakage, temporal attention is only allowed to attend to historical time steps from the current prediction state, and its calculation is given by:

(27) $$T_{i}=\mathrm{Softmax}\left(\frac{Q_{i}K_{i}^{⊤}+M}{\sqrt{C_{o}}}\right)V_{i},\;$$

where $Q_{i}=P_{i}W_{Q}$, $K_{i}=P_{i}W_{K}$, $V_{i}=P_{i}W_{V}$, and M denotes the causal temporal mask matrix used to mask non-historical temporal positions. This design enables the model to jointly capture morning and evening peak periodicity, short-term congestion mutations, and non-stationary changes caused by sudden events.

The multi-task prediction layer maps the temporal encoding results to traffic states over multiple future prediction horizons. Let the prediction horizon set be denoted as Ω. Speed, traffic flow, congestion level, and risk score are produced by different task-specific heads:

(28) $${\hat{Y}}_{\omega }^{r}=T_{:,L}W_{\omega }^{r}+b_{\omega }^{r},\;{\hat{Y}}_{\omega }^{c}=\mathrm{Softmax}\left(T_{:,L}W_{\omega }^{c}+b_{\omega }^{c}\right),$$

where ${\hat{Y}}_{\omega }^{r}$ denotes the continuous traffic state prediction at future horizon ω, and ${\hat{Y}}_{\omega }^{c}$ denotes the congestion level classification result. The training objective is composed of regression loss, classification loss, and risk ranking loss:

(29) $$L_{p}=\sum_{\omega \in \Omega }\left(\mu_{1}{\left|{\hat{Y}}_{\omega }^{r}-Y_{\omega }^{r}\right|}_{1}+\mu_{2}\mathrm{CE}\left({\hat{Y}}_{\omega }^{c},Y_{\omega }^{c}\right)+\mu_{3}L_{rank}\right).$$

To make the prediction results interpretable, a causal contribution score is further defined to measure the influence of historical nodes, sensing modalities, and external disturbances on the prediction result of the target node. For target node $v_{j}$, the explanatory contribution from source node $v_{i}$ is defined as:

(30) $$\Gamma_{ij}^{\omega }=\sum_{t=1}^{L}\left|\frac{\partial {\hat{y}}_{j,\omega }}{\partial P_{i,t}}\right|\cdot A_{ij,t}^{c}\cdot {\left|P_{i,t}\right|}_{2}.$$

This formula simultaneously considers prediction gradient sensitivity, dynamic causal edge weight, and source node state intensity, thereby avoiding the instability caused by explanations based solely on attention weights. Furthermore, if the fusion weight of the *m*-th sensing modality for the node representation is denoted as $\alpha_{i,t}^{m}$, the modality-level contribution can be expressed as:

(31) $$\Psi_{m}^{\omega }=\sum_{i,j,t}\alpha_{i,t}^{m}\Gamma_{ij}^{\omega }.$$

Accordingly, the model can output key congestion source road segments, major propagation chains, and the relative contributions of traffic flow, trajectories, weather, events, and visual modalities.

For operational interpretation, candidate propagation paths are extracted by tracing directed edges with high dynamic edge weights and high contribution scores from upstream source nodes to the target node. A path is retained only when its edges are consistent with the directed road topology, satisfy temporal precedence through the lag parameter δ, and make a positive contribution to the target prediction. The resulting path is accompanied by its source-node contribution, edge strength, dominant sensing modality, and associated external disturbance. These quantities allow traffic operators to verify whether the reported pathway is supported by upstream state degradation, event information, and physically feasible road connectivity, and to identify the road segments at which intervention may be most effective.

The rationality of this explanation mechanism can be illustrated by first-order Taylor expansion. For the target prediction function f(·), when a small perturbation $\Delta P_{i,t}$ occurs in the source node representation, the prediction change can be approximated as:

(32) $$\Delta {\hat{y}}_{j,\omega }\approx \frac{\partial f}{\partial P_{i,t}}\Delta P_{i,t}.$$

When this perturbation is located on a strong causal edge $A_{ij,t}^{c}$, its explanatory significance for the target congestion prediction becomes stronger. Therefore, $\Gamma_{ij}^{\omega }$ can jointly characterize the influence source from the perspectives of prediction sensitivity and causal propagation strength.

In this formulation, gradient sensitivity alone is not treated as evidence of a propagation relationship. A source node is considered relevant only when its gradient contribution is supported simultaneously by a strong directed edge, temporal precedence, and the topology- and event-conditioned graph structure. This joint criterion provides a model-internal conditional verification mechanism and avoids generating actionable paths solely from attention scores or instantaneous correlations.

When applied to the proposed task, this module can not only output future speed, traffic flow, and congestion levels, but also explain whether a specific prediction result is caused by upstream road speed degradation, accident event propagation, enhanced rainfall influence, or increased public transportation pressure. Thus, a complete explanation chain of prediction result, key road segments, propagation paths, and triggering factors is formed. Compared with ordinary graph-temporal prediction models, this design can improve long-horizon prediction capability and provide more intuitive decision-making support for signal timing control, route guidance, accident handling, and public transportation dispatching.

## 4. Results and Discussion

### 4.1. Experimental Configuration

#### 4.1.1. Hardware and Software Platforms

The experiments were conducted on a deep learning server equipped with an Intel Xeon Silver processor, 128 GB of DDR4 memory, and one NVIDIA RTX 4090 GPU with 24 GB of memory. The operating system was Ubuntu 22.04, and the model was implemented using PyTorch 2.0, CUDA 11.8, cuDNN 8.9, and PyTorch Geometric 2.3. Data preprocessing was performed using NumPy 1.24, Pandas 2.0, and Scikit-learn 1.2. All experiments were conducted using mixed-precision training, and the random seeds of Python 3.10, NumPy, and PyTorch were fixed to 2024. Deterministic CUDA operations were enabled whenever supported. The dataset was first divided chronologically into training, validation, and testing periods with proportions of 70%, 15%, and 15%, respectively. The final test period was held out throughout model development and was used only once for reporting the final performance. Within the initial 70% training period, a five-fold rolling-origin validation strategy was further adopted for hyperparameter selection and stability analysis. In each fold, the training window always preceded the corresponding validation window in time, and the training set was progressively expanded without using any observations from future periods.

The historical input length was set to L=12, corresponding to the previous 60 min under the unified 5-min sampling interval. The prediction horizons were 15, 30, and 60 min. The common latent feature dimension was d=64; the batch size was 32, and the maximum number of training epochs was 200. The Adam optimizer was used with an initial learning rate of $1\times 10^{-3}$ and a weight decay of $1\times 10^{-5}$. The learning rate was updated using cosine annealing with $T_{\max}=200$ and a minimum learning rate of $1\times 10^{-5}$. Gradient clipping was applied with a maximum norm of 5.0. Early stopping was activated when the validation loss did not improve for 20 consecutive epochs. For the roadside-camera visual encoder, eight frames were uniformly sampled from each 5-min interval and resized to 224×224. A ResNet-50 backbone pretrained on ImageNet-1K was used to extract frame-level visual representations. The first three residual stages were frozen, while the final residual stage was fine-tuned using the traffic-camera images. The 2048-dimensional global-average-pooled feature was projected to 256 dimensions through a fully connected layer and then mapped to the common 64-dimensional latent space. Frame-level features within each time window were aggregated using a two-layer temporal attention encoder with four attention heads, a feed-forward dimension of 256, and a dropout rate of 0.1. For traffic flow, GPS trajectory, and public transportation sequences, each modality-specific encoder consisted of one one-dimensional convolutional layer with 64 output channels, a kernel size of 3, and ReLU activation, followed by a single-layer GRU with a hidden dimension of 64. Weather and traffic-event variables were first embedded into 32-dimensional representations and then projected to 64 dimensions. Layer normalization was applied to the output of each modality-specific encoder. The reliability-aware fusion module used an eight-head cross-modal attention mechanism with a model dimension of 64, a feed-forward dimension of 256, and a dropout rate of 0.1. The modality reliability estimator was implemented as a two-layer multilayer perceptron with hidden dimensions of 64 and 32, followed by a sigmoid output layer. Its inputs included the modality representation, missing-data mask, sensing-quality indicator, temporal-consistency score, and spatial-consistency score. The reliability adjustment coefficient in the attention logits was set to λ=0.5. In the dynamic spatiotemporal causal graph learning module, three parallel temporal convolution branches with kernel sizes {2,3,6} were used, and each branch contained 64 output channels. Their concatenated features were projected to a graph representation dimension of $C_{g}=128$. The source and target edge-projection dimensions were both set to $C_{a}=64$. Candidate temporal lags were selected from δ∈{1,2,…,6}, corresponding to 5–30 min. For each node, the ten highest-scoring candidate neighbors were retained to construct the sparse dynamic graph. The topology-prior coefficient was set to η=0.5, the graph sparsity coefficient was set to $\beta =1\times 10^{-4}$, and the temporal smoothness coefficient was set to $\gamma =1\times 10^{-3}$. The congestion prediction module contained two dynamic graph propagation blocks and two causal temporal Transformer layers. Each graph propagation block used a hidden dimension of 128 and a dropout rate of 0.1. Each Transformer layer contained eight attention heads, a hidden dimension of 128, a feed-forward dimension of 256, and a dropout rate of 0.1. The regression, classification, and risk-ranking loss weights were set to $\mu_{1}=1.0$, $\mu_{2}=1.0$, and $\mu_{3}=0.1$, respectively. The graph-learning loss was combined with the prediction loss using a coefficient of 0.1.

#### 4.1.2. Baseline Models and Evaluation Metrics

To comprehensively evaluate the proposed framework, baseline models were selected from several representative categories, including traditional statistical models, machine learning methods, temporal deep learning models, spatiotemporal graph neural networks, multimodal fusion approaches, causal graph learning methods, and recent state-of-the-art traffic prediction models. Specifically, ARIMA [51], XGBoost [52], LSTM [53], Transformer [54], STGCN [55], Graph WaveNet [56], GMAN [57], Multimodal Transformer [58], Causal Temporal Graph Network [59], DST2former [60], TDMGCN [61], and MGSGDM [36] were included.

ARIMA and XGBoost represent traditional statistical and machine learning approaches for modeling temporal patterns and nonlinear feature relationships, respectively. LSTM and Transformer were selected as representative temporal deep learning models. STGCN and Graph WaveNet were adopted to evaluate fixed-topology and adaptive graph-based spatiotemporal modeling, while GMAN was selected as an attention-based graph learning method. Multimodal Transformer was used to compare multimodal feature fusion capability. Causal Temporal Graph Network was included as a representative causal graph learning method. In addition, three recently published methods were introduced for comparison. DST2former [60] models dynamic traffic trends through adaptive temporal-spatial feature fusion, TDMGCN [61] combines Transformer-based temporal modeling with graph convolution for traffic prediction, and MGSGDM [36] utilizes multi-granularity graph diffusion to capture complex mobility patterns. These recent methods provide additional comparisons with respect to dynamic dependency learning, graph representation, and temporal modeling.

Although the above methods improve traffic prediction from different perspectives, most existing approaches mainly focus on temporal dependency modeling or correlation-based spatial interaction learning. In contrast, the proposed framework further incorporates multisource sensing fusion, reliability-aware modality weighting, dynamic causal graph construction, and causal propagation explanation, aiming to improve both prediction performance and interpretability.

For fair comparison, official implementations or author-released codes were adopted whenever available. All neural baselines were retrained under the same chronological data partition, input window, prediction horizon, preprocessing pipeline, and early-stopping criterion. Single-source models, including ARIMA, XGBoost, LSTM, Transformer, STGCN, Graph WaveNet, and GMAN, used common traffic-state features, while multimodal methods used all available sensing modalities. Hyperparameters were selected according to validation-set MAE. For neural models, the learning rate, hidden dimension, number of layers, and dropout were searched from $\left\{10^{-4},5\times 10^{-4},10^{-3}\right\}$, {64,128,256}, {1,2,3}, and {0.1,0.3,0.5}, respectively. For traditional models, ARIMA parameters (p,d,q) were searched within {0,1,2,3}, while XGBoost parameters were selected from predefined estimator, depth, and learning-rate ranges. The best configuration on the validation set was used for final testing.

In terms of evaluation metrics, MAE, RMSE, and MAPE were used to evaluate continuous traffic state prediction, including speed and flow estimation. Accuracy, Precision, Recall, F1-score, and AUC were adopted for congestion classification. For congestion early warning, CDR and FAR were further considered. In addition, causal edge stability, key road segment identification accuracy, and event impact consistency were used to evaluate the explanation capability of the proposed framework.

(33) $$MAE=\frac{1}{N}\sum_{i=1}^{N}\left|y_{i}-{\hat{y}}_{i}\right|,$$

(34) $$RMSE=\sqrt{\frac{1}{N}\sum_{i=1}^{N}{\left(y_{i}-{\hat{y}}_{i}\right)}^{2}},$$

(35) $$MAPE=\frac{100\%}{N}\sum_{i=1}^{N}\left|\frac{y_{i}-{\hat{y}}_{i}}{y_{i}}\right|,$$

(36) $$Accuracy=\frac{TP+TN}{TP+TN+FP+FN},$$

(37) $$Precision=\frac{TP}{TP+FP},\;Recall=\frac{TP}{TP+FN},$$

(38) $$F1=2\times \frac{Precision\times Recall}{Precision+Recall},$$

(39) $$FAR=\frac{FP}{FP+TN},\;CDR=\frac{TP}{TP+FN}.$$

Here, $y_{i}$ and ${\hat{y}}_{i}$ denote the ground-truth and predicted traffic states, respectively. TP, TN, FP, and FN represent true positives, true negatives, false positives, and false negatives. AUC evaluates the overall discrimination capability of congestion classification, while causal edge stability measures the consistency of learned dynamic graph structures across different time windows.

### 4.2. Traffic State Prediction Task

This experiment was designed to systematically evaluate the performance differences among different types of models in multi-horizon urban traffic state prediction tasks and to verify the effectiveness of the proposed method in short-term, medium-term, and long-term prediction scenarios.

**Figure 4:**
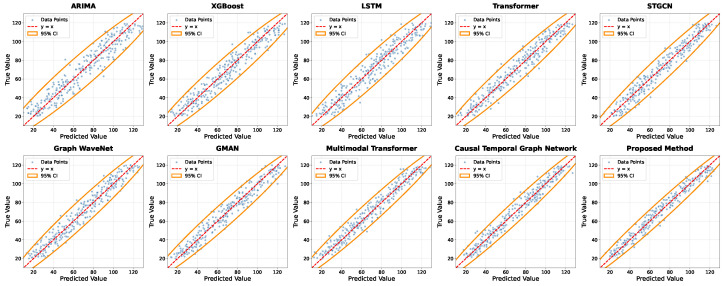
Comparison of predicted and actual traffic speeds on representative urban road segments, showing model performance over varying time horizons.

Table 2 and Figure 4 report the MAE, RMSE, and MAPE results under three prediction horizons, namely 15 min, 30 min, and 60 min. From the overall trend, the prediction errors of all models increase as the prediction horizon is extended from 15 min to 60 min, indicating that traffic state prediction becomes significantly more difficult as the forecasting step increases. ARIMA shows the weakest performance across all three prediction horizons, with an MAE of 8.26 and a MAPE of 25.91% under the 60-min prediction horizon, indicating that linear autoregressive structures are insufficient for characterizing nonlinear variations in complex traffic systems. XGBoost achieves a clear improvement over ARIMA, suggesting that tree-based nonlinear feature partitioning can better exploit multisource traffic features. However, because continuous temporal dependency modeling is absent, relatively large errors still occur in long-horizon prediction. LSTM and Transformer further outperform traditional models, with Transformer achieving an MAE of 6.42 under the 60-min horizon, which is lower than the 6.94 obtained by LSTM. This result indicates that the self-attention mechanism is more effective in capturing long-range temporal dependencies. STGCN, Graph WaveNet, and GMAN further reduce prediction errors compared with pure temporal models, demonstrating that road topology and spatial dependencies play an important role in traffic state prediction. Among them, GMAN consistently outperforms STGCN and Graph WaveNet across the three prediction horizons, indicating that the multi-attention mechanism has stronger representational capability for global spatiotemporal dependencies.

From the perspective of model mechanisms, the proposed method achieves the best results on all metrics, with MAE values of 3.21, 3.79, and 4.48 under the 15-min, 30-min, and 60-min prediction horizons, respectively. Compared with Causal Temporal Graph Network, further reductions are achieved, indicating that the proposed method not only inherits the advantage of causal graph models in propagation relationship modeling, but also improves traffic state representation under complex scenarios through multisource sensing collaborative encoding and dynamic causal adjacency learning. The results of Multimodal Transformer are better than those of GMAN, indicating that multimodal information, including traffic flow, trajectories, images, weather, and events, can supplement external disturbance factors that are difficult to cover using single-source traffic sequences. However, Multimodal Transformer mainly learns correlations through cross-modal attention and lacks explicit constraints on causal propagation directions among road segments. Causal Temporal Graph Network further improves upon multimodal methods, suggesting that causal relationship modeling helps alleviate error accumulation caused by static correlations. Nevertheless, this model usually provides insufficient consideration of sensor reliability and event-conditioned changes, and therefore certain errors remain under long prediction horizons. In the proposed method, low-quality modality noise is suppressed through reliability-aware fusion, congestion propagation intensity under different temporal and event conditions is characterized through a dynamic spatiotemporal causal graph, and spatial diffusion and temporal evolution are jointly modeled through a graph-temporal prediction structure. Therefore, low local errors can be maintained in short-term prediction, error propagation can be mitigated in long-term prediction, and stronger prediction accuracy, stability, and adaptability to complex traffic scenarios can be achieved.

### 4.3. Congestion Identification and Early Warning

This experiment was designed to verify the congestion identification capability and early warning reliability of different models under complex traffic scenarios. In particular, classification performance and false alarm control were evaluated under three typical non-stationary scenarios: peak hours, rainy weather, and traffic events. Unlike ordinary traffic state prediction tasks, congestion identification and early warning focus more on whether a model can accurately identify congestion states that are about to occur or have already formed while reducing missed alarms and controlling the false alarm rate.

**Figure 5:**
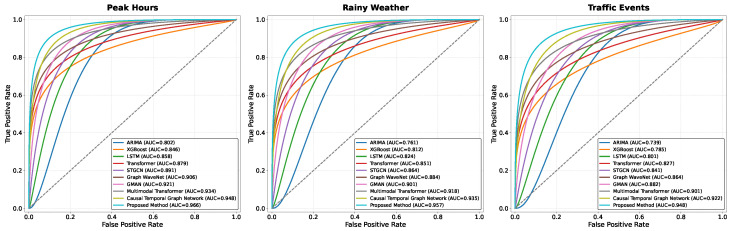
ROC curves for congestion classification across different models, demonstrating the proposed method’s superior detection capability.

As shown in Table 3 and Figure 5, ARIMA exhibits the weakest performance across the three scenarios. In the traffic event scenario, its F1 and AUC are only 0.652 and 0.739, respectively, while the FAR reaches 0.261. This indicates that linear time-series models are difficult to adapt to traffic state mutations caused by sudden events and complex external disturbances. XGBoost achieves a clear improvement over ARIMA, with an F1 of 0.781 during peak hours, indicating that tree-based nonlinear partitioning can use multisource input features to identify congestion to a certain extent. However, because continuous temporal dependency and spatial propagation modeling are absent, relatively high false alarm rates still occur under rainy weather and traffic event scenarios. LSTM and Transformer further improve identification performance. Transformer achieves higher F1 values than LSTM in all three scenarios, reaching 0.752 in the traffic event scenario, suggesting that the self-attention mechanism is more suitable than recurrent structures for capturing state changes and contextual dependencies over longer time ranges. Nevertheless, both models mainly describe traffic states along the temporal dimension and lack explicit modeling of spatial diffusion relationships in road networks. Therefore, key interregional coupled changes cannot be sufficiently captured in complex scenarios with strong congestion propagation.

Further comparisons among spatiotemporal graph models show that STGCN, Graph WaveNet, and GMAN significantly outperform pure temporal models, indicating that the introduction of road topology and spatial dependency modeling helps improve congestion identification and early warning performance. Graph WaveNet outperforms STGCN, suggesting that adaptive adjacency matrices can compensate for the limitations of fixed road topology under complex traffic scenarios. GMAN achieves F1 values of 0.869, 0.832, and 0.814 under peak hours, rainy weather, and traffic events, respectively, demonstrating that graph attention mechanisms can better capture the importance of different regions and time steps. Multimodal Transformer further improves upon GMAN, indicating that multisource heterogeneous information is valuable for congestion early warning in complex scenarios. Weather, event, trajectory, and visual features can supplement external disturbance factors that are difficult to express using single-source traffic flow data. Causal Temporal Graph Network continues to improve performance in all three scenarios, indicating that causal dependency modeling helps distinguish ordinary correlations from real propagation effects, thereby reducing false alarm rates. The proposed method achieves the best results in all scenarios, with F1 values of 0.927, 0.904, and 0.893 and AUC values of 0.966, 0.957, and 0.948 under peak hours, rainy weather, and traffic events, respectively. Meanwhile, FAR is reduced to 0.061, 0.072, and 0.081, respectively. From the perspective of mathematical modeling characteristics, low-quality sensor noise is suppressed through reliability-aware multimodal fusion, directional propagation relationships among roads under different scenarios are characterized through dynamic spatiotemporal causal graph learning, and consistency among key events, key road segments, and congestion outcomes is strengthened through causal explanation constraints. Therefore, true congestion risks and short-term random fluctuations can be more accurately distinguished under peak traffic superposition, rainfall disturbance, and sudden event impacts, resulting in higher identification accuracy, stronger discriminative capability, and lower false alarm rates.

### 4.4. Ablation Study

This experiment was designed to verify the effectiveness of each key component in the proposed spatiotemporal causal graph learning framework based on multisource urban sensing data and to analyze the contributions of different modality information, fusion mechanisms, dynamic graph structures, temporal modeling, and causal explanation constraints to final traffic prediction and congestion identification performance.

**Figure 6:**
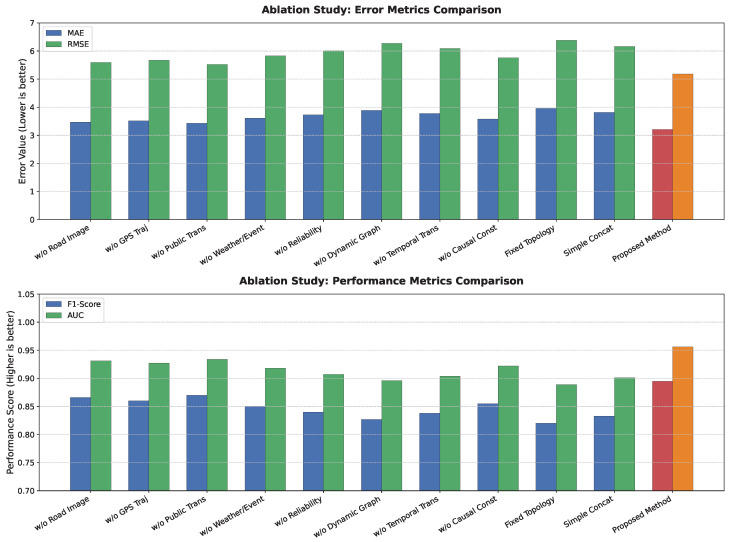
Ablation study results highlighting the contribution of each module to overall traffic prediction accuracy, F1 score, and AUC.

As shown in Table 4 and Figure 6, the complete model achieves the best performance across all metrics, with an MAE of 3.21, an F1 score of 0.895, and an AUC of 0.956. Removing individual sensing modalities leads to consistent performance degradation, confirming that they provide complementary information. Road image features improve the representation of vehicle accumulation, lane occupancy, queue formation, and abnormal stopping, while GPS trajectory features contribute to movement direction, travel-time variation, route transfer, and regional travel demand. Public transportation data provide additional information around transport hubs and high-demand corridors. Weather and event features have a stronger effect than the other auxiliary modalities: removing them increases the MAE to 3.61 and reduces the AUC to 0.918, indicating that rainfall, accidents, construction, and traffic control are important for distinguishing recurrent congestion from externally induced abnormal states.

The fusion and temporal modeling components also make substantial contributions. Removing the reliability-aware fusion module increases the MAE to 3.73 and reduces the F1 score to 0.840, while replacing attention fusion with simple concatenation further increases the MAE to 3.82. These results show that multimodal features should not be treated as equally reliable, because their quality varies with missing data, sensor noise, and synchronization conditions. The reliability-aware attention mechanism adjusts modality contributions according to sensing quality and contextual consistency. Removing the Temporal Transformer increases the MAE to 3.78, demonstrating that graph propagation alone cannot fully capture peak-hour periodicity, delayed congestion effects, and long-range temporal dependencies.

The largest performance degradation is associated with graph structure modeling. Removing dynamic causal graph learning increases the MAE to 3.89 and reduces the F1 score to 0.827, whereas replacing it with a fixed topology graph produces the worst result, with an MAE of 3.96 and an AUC of 0.889. This indicates that physical road connectivity is a useful prior but cannot fully describe time-varying congestion propagation under peak demand, rainfall, accidents, construction, and route diversion. The dynamic graph enables the model to adjust the direction and strength of road interactions according to the current traffic context. In addition, removing the causal explanation constraint reduces the AUC and CDR to 0.922 and 0.849, respectively, showing that this constraint not only supports interpretation but also regularizes the graph by aligning lagged source-node information, dynamic edge weights, and future target-state predictions. Overall, the ablation results indicate that multisource sensing provides complementary observations, reliability-aware fusion controls modality quality, and the dynamic causal graph forms the main source of improvement in both prediction and congestion identification.

### 4.5. Discussion

The experimental results demonstrate that the proposed method has strong practical application value in urban traffic congestion prediction and early warning tasks. In high-density urban traffic scenarios such as Shanghai, Hangzhou, and Beijing, commuting flows often converge rapidly at expressway entrances, river-crossing bridges, school surroundings, and large commercial districts during morning and evening peak hours. Once the speed of a local road segment decreases, adjacent arterial roads and detour roads may also be affected. Traditional methods usually extrapolate only from historical speed degradation trends and tend to issue warnings after congestion has already formed. By contrast, the proposed method can jointly consider travel demand changes from GPS trajectories, queue states from roadside cameras, passenger flow pressure at bus stops, and road topology propagation relationships, thereby identifying key road segments that may become congestion sources in advance. For example, in holiday scenarios around the West Lake scenic area in Hangzhou, increased parking demand, rising passenger flow at bus stops, and declining speeds on surrounding roads often occur simultaneously. Through multisource sensing data fusion, the proposed method can identify such compound congestion risks rather than rely only on the speed variation of a single road segment.

The advantages of the proposed method become more evident in sudden accident and adverse weather scenarios. Taking an accident on an expressway ramp as an example, vehicle queues may first appear near the accident location, followed by a decrease in upstream entrance capacity. Some vehicles may then divert to adjacent ground roads, causing local congestion to spread to surrounding areas. The dynamic spatiotemporal causal graph in the proposed method can adaptively enhance causal propagation relationships among related road segments according to accident location, road direction, historical traffic states, and detour path changes, thereby enabling more accurate judgment of congestion diffusion directions. Under heavy rainfall or low-visibility conditions, camera image quality may decrease, and some GPS trajectories may become sparse or drift. However, the reliability-aware fusion mechanism can reduce the weights of low-quality modalities and rely more on traffic flow detectors, road environmental sensors, and neighboring node states for inference. Therefore, the proposed method can not only output future traffic states, but also explain whether congestion is caused by accident propagation, rainfall effects, increased public transportation pressure, or concentrated regional travel demand. This analysis pattern of prediction results, congestion sources, propagation paths, and inducing factors better meets the practical needs of traffic management departments and can be used for signal timing adjustment, traffic guidance release, bus dispatch optimization, and emergency response priority determination.

**Figure 7:**
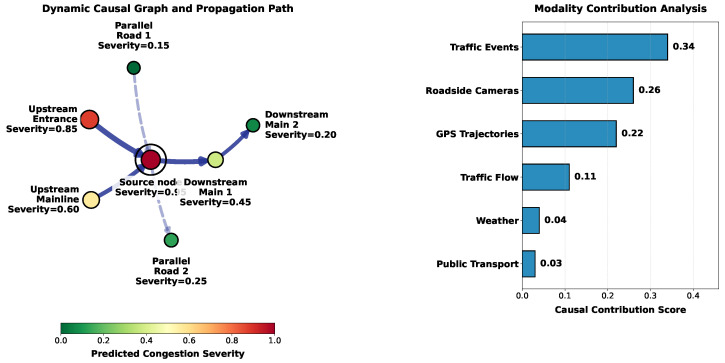
Visualization of congestion source identification and causal propagation paths. The highlighted nodes indicate detected congestion sources; directed edges represent learned causal propagation relationships, and modality contribution scores illustrate the influence of different sensing sources.

To further illustrate the explainability capability of the proposed framework, a case analysis is conducted based on a congestion propagation event. As shown in Figure 7, the model identifies road segment $v_{i}$ as the primary congestion source and detects the propagation path from upstream road segments to downstream affected areas. The thickness of the causal edges represents the learned propagation intensity, while the node colors indicate the predicted congestion severity. The results show that the congestion influence is not simply determined by physical adjacency; instead, the model assigns higher causal weights to upstream roads with consistent temporal precedence and stronger contribution to future congestion states. For example, during an accident scenario, the upstream entrance road receives a higher causal contribution score than nearby parallel roads, indicating that the model distinguishes the actual congestion-triggering segment from roads that are only geographically adjacent. Furthermore, the modality contribution analysis shows that different sensing sources provide complementary explanations for congestion formation. In this case, GPS trajectory features contribute mainly to identifying travel-demand redistribution, roadside camera features provide information related to queue formation and lane occupancy changes, and event features increase the contribution of accident-related propagation paths. By combining causal edge weights and modality-level contribution scores, the proposed method generates an interpretable chain consisting of congestion source identification, propagation direction estimation, and external factor attribution. Such visualized explanations can help traffic operators understand not only where congestion occurs, but also why congestion develops and which upstream locations should be prioritized for intervention.

From the perspective of computational complexity, the proposed method avoids constructing and propagating over an unrestricted dense graph during each prediction step. After candidate-neighbor sampling, each node retains at most k=10 candidate relationships, so the graph-generation and graph-propagation costs are approximately $O(LNkC_{g})$ rather than $O(LN^{2}C_{g})$, where *N* is the number of road nodes, *L* is the historical sequence length, and $C_{g}$ is the graph hidden dimension. The temporal attention component has a complexity of approximately $O(NSL^{2}C_{o})$, where *S* denotes the number of temporal attention layers. Since the historical window is fixed at L=12, the main computation grows approximately linearly with the number of nodes and retained graph edges. Under the experimental configuration of approximately 3200 road nodes, an input window of 12 time steps, and 10 sampled neighbors per node, the average model inference time was approximately 38 ms per prediction window on one NVIDIA RTX 4090 GPU. Including feature loading, graph construction, multimodal fusion, and output generation, the end-to-end processing time was approximately 0.11 s per window. This latency is substantially shorter than the 5-min data-update interval and therefore supports online traffic-state prediction and warning generation.

Regarding generalization, the model does not depend solely on fixed road identifiers or a single traffic pattern. The shared modality encoders learn common representations of traffic flow, trajectory movement, visual congestion, public transportation pressure, weather, and event disturbances, while the dynamic graph is regenerated according to the current node states and external context. This design allows the propagation structure to change across weekdays, weekends, peak periods, adverse weather, and traffic-event scenarios. In addition, chronological data partitioning, rolling-origin validation, modality masking, dropout, weight decay, and early stopping reduce the risk of fitting only the traffic patterns of a specific period. Nevertheless, transferring the model to another city may still require recalibration of the road topology, sensor distributions, and modality-quality indicators. The shared encoding and dynamic graph parameters can be retained as initialization, while a limited amount of local traffic data can be used for fine-tuning.

### 4.6. Limitation and Future Work

Certain limitations still remain in this study. First, the experimental data were mainly obtained from the road network in the main urban area of Hangzhou. Although multiple scenarios were covered, including arterial roads, expressways, commercial districts, residential areas, school surroundings, and transportation hubs, differences still exist among different cities in terms of road structure, travel habits, signal control strategies, and public transportation systems. Therefore, the generalization capability of the model in other cities still needs to be further verified. Second, multisource data, including traffic flow, GPS trajectories, road images, public transportation, weather, and events, were integrated in this study. However, in real traffic systems, some sensing devices may suffer from long-term missing data, positioning errors, image occlusion, or incomplete event records, which may affect the model’s judgment of complex congestion causes. Although the reliability-aware fusion mechanism can alleviate some data quality problems, prediction instability may still occur under extreme sensor failure scenarios. Future research can be further extended in three directions. First, multi-city data can be introduced for cross-regional transfer learning and domain-adaptive modeling so that the generalization capability of the model under different road networks and traffic organization patterns can be improved. Second, real-time signal timing, road control strategies, bus dispatching schemes, parking lot states, and other management-side data can be further incorporated so that congestion can not only be predicted, but the potential effects of different traffic intervention measures can also be evaluated. Third, the proposed framework can be deployed on edge computing devices or traffic management platforms to explore practical applications, such as low-latency online prediction, real-time congestion source identification, and dynamic route guidance, thereby further enhancing its engineering value in intelligent traffic governance.

## 5. Conclusions

A spatiotemporal causal graph learning framework based on multisource urban sensing data is proposed in this study to address the demands of traffic congestion prediction and intelligent sensing in smart cities. Urban traffic congestion is not merely a traffic state change on an individual road segment, but a complex spatiotemporal propagation process jointly driven by traffic flow, vehicle trajectories, road visual states, public transportation pressure, meteorological conditions, and sudden events. To address the insufficient utilization of multisource sensing data, the difficulty of static road topology in characterizing dynamic congestion propagation relationships, and the lack of interpretability in prediction results in existing methods, a multimodal modeling framework for urban traffic state perception is constructed. A multisource urban sensing data collaborative encoding module, a dynamic spatiotemporal causal graph learning module, and a causality-explanation-driven congestion prediction module are designed, thereby realizing a complete modeling process from heterogeneous sensing signal fusion and dynamic propagation relationship learning to explainable traffic state prediction. The proposed method can adaptively adjust fusion weights according to the reliability of different sensing modalities and further learn time-varying causal propagation relationships from historical traffic states, road topology, and external disturbances, thereby more accurately characterizing the formation, diffusion, and evolution mechanisms of congestion. The experimental results verify the advantages of the proposed method in traffic state prediction, congestion identification and early warning, and module effectiveness. In the traffic state prediction task, the proposed method achieves MAE values of 3.21, 3.79, and 4.48 under the 15-min, 30-min, and 60-min prediction horizons, respectively, outperforming comparison models such as ARIMA, XGBoost, LSTM, Transformer, STGCN, Graph WaveNet, GMAN, Multimodal Transformer, and the Causal Temporal Graph Network. In the congestion identification and early warning task under complex scenarios, F1 values of 0.927, 0.904, and 0.893 are obtained under peak-hour, rainy-weather, and traffic-event scenarios, respectively; the corresponding AUC values reach 0.966, 0.957, and 0.948; and the FAR values are reduced to 0.061, 0.072, and 0.081, indicating that strong discriminative capability and low false alarm rates can be maintained under non-stationary traffic conditions. The ablation study further shows that the complete model achieves an Accuracy of 0.914, a Precision of 0.902, a Recall of 0.889, an F1 of 0.895, and an AUC of 0.956, confirming that multisource sensing fusion, reliability-aware attention, dynamic causal graph modeling, and causal explanation constraints all make important contributions to the final performance. Overall, an effective technical pathway is provided by the proposed method for AI-driven urban traffic sensing, congestion risk identification, and intelligent traffic governance.

## Figures and Tables

**Table 1:** Statistics of multisource urban traffic sensing data and collection devices.

Data Type	Collection Sensor Model	Quantity
Traffic flow data	RTMS Sx-300 microwave traffic flow detector; Sensys Networks VDS240 geomagnetic vehicle detector	Approximately 12,500,000 records
GPS trajectory data	Quectel L76-L GNSS positioning module; u-blox NEO-M8N GPS receiver module	Approximately 38,000,000 trajectory points
Road image data	Hikvision DS-2CD7A47G0/P-IZHS roadside surveillance camera; Dahua ITC431-RW1F-IRL8 intelligent traffic camera	Approximately 860,000 image frames
Public transportation data	Teltonika FMB920 vehicle-mounted positioning terminal; bus IC card automatic fare collection system and station passenger flow counter	Approximately 4,200,000 records
Meteorological data	Vaisala WXT536 multi-parameter weather sensor; Davis Vantage Pro2 automatic weather station	Approximately 52,000 records
Traffic event data	Traffic management platform event recording terminal; Hikvision iDS-TCD403-BI intelligent event detection camera	Approximately 18,000 event records
Road topology data	Trimble R12 GNSS surveying receiver; urban road GIS collection terminal	Approximately 3200 nodes and 8600 edges

**Table 2:** Performance comparison of different models on the traffic state prediction task (mean ± standard deviation over five runs).

Method	15 min			30 min			60 min			*p*-Value
	MAE	RMSE	MAPE (%)	MAE	RMSE	MAPE (%)	MAE	RMSE	MAPE (%)	
ARIMA	5.72±0.083	8.91±0.124	18.64±0.216	6.83±0.097	10.42±0.156	21.37±0.284	8.26±0.112	12.85±0.193	25.91±0.331	<0.001
XGBoost	4.96±0.071	7.82±0.109	16.58±0.193	5.87±0.084	9.24±0.132	18.96±0.241	7.18±0.102	11.36±0.167	22.71±0.295	<0.001
LSTM	4.71±0.065	7.46±0.103	15.93±0.182	5.62±0.078	8.87±0.121	18.32±0.225	6.94±0.091	10.98±0.154	21.85±0.271	<0.001
Transformer	4.43±0.061	7.02±0.096	14.93±0.174	5.26±0.071	8.31±0.113	17.18±0.213	6.42±0.084	10.15±0.143	20.38±0.248	<0.001
STGCN	4.21±0.058	6.73±0.091	14.36±0.161	5.03±0.067	8.05±0.105	16.59±0.201	6.18±0.079	9.82±0.136	19.71±0.232	<0.001
Graph WaveNet	4.02±0.054	6.41±0.087	13.76±0.154	4.81±0.063	7.68±0.098	15.87±0.187	5.89±0.073	9.41±0.129	18.75±0.221	<0.001
GMAN	3.89±0.051	6.22±0.083	13.21±0.148	4.65±0.059	7.39±0.094	15.29±0.176	5.62±0.069	8.96±0.122	17.84±0.214	<0.001
Multimodal Transformer	3.63±0.046	5.84±0.078	12.36±0.137	4.31±0.053	6.91±0.087	14.18±0.162	5.23±0.064	8.39±0.116	16.56±0.198	<0.001
Causal Temporal Graph Network	3.49±0.043	5.61±0.074	11.82±0.132	4.11±0.050	6.59±0.083	13.47±0.155	4.91±0.059	7.92±0.109	15.64±0.187	<0.001
DST2former	3.38±0.041	5.45±0.072	11.51±0.128	3.98±0.047	6.40±0.079	13.06±0.149	4.76±0.056	7.65±0.103	15.18±0.181	<0.001
TDMGCN	3.34±0.039	5.39±0.070	11.38±0.126	3.94±0.046	6.34±0.077	12.91±0.146	4.69±0.054	7.54±0.101	14.96±0.178	<0.001
MGSGDM	3.30±0.037	5.32±0.068	11.21±0.123	3.87±0.044	6.25±0.075	12.74±0.142	4.59±0.052	7.43±0.097	14.72±0.172	<0.001
Proposed Method	** 3.21±0.031 **	** 5.18±0.061 **	** 10.94±0.112 **	** 3.79±0.038 **	** 6.13±0.068 **	** 12.52±0.131 **	** 4.48±0.046 **	** 7.31±0.089 **	** 14.36±0.159 **	–

**Table 3:** Performance comparison of congestion identification and early warning under different complex traffic scenarios (mean ± standard deviation over five runs).

Method	Peak Hours			Rainy Weather			Traffic Events			*p*-Value
	F1	AUC	FAR	F1	AUC	FAR	F1	AUC	FAR	
ARIMA	0.731±0.014	0.802±0.017	0.196±0.009	0.684±0.018	0.761±0.021	0.238±0.012	0.652±0.020	0.739±0.022	0.261±0.014	<0.001
XGBoost	0.781±0.012	0.846±0.015	0.162±0.008	0.736±0.015	0.812±0.018	0.198±0.010	0.704±0.017	0.785±0.019	0.221±0.011	<0.001
LSTM	0.796±0.011	0.858±0.014	0.153±0.007	0.751±0.014	0.824±0.016	0.187±0.009	0.721±0.015	0.801±0.017	0.209±0.010	<0.001
Transformer	0.819±0.010	0.879±0.012	0.137±0.007	0.776±0.012	0.851±0.015	0.168±0.008	0.752±0.014	0.827±0.015	0.188±0.009	<0.001
STGCN	0.834±0.009	0.891±0.011	0.126±0.006	0.792±0.011	0.864±0.013	0.156±0.007	0.768±0.012	0.841±0.014	0.176±0.008	<0.001
Graph WaveNet	0.852±0.009	0.906±0.010	0.113±0.006	0.813±0.010	0.884±0.012	0.141±0.007	0.792±0.011	0.864±0.012	0.158±0.007	<0.001
GMAN	0.869±0.008	0.921±0.010	0.101±0.005	0.832±0.009	0.901±0.011	0.127±0.006	0.814±0.010	0.882±0.011	0.143±0.007	<0.001
Multimodal Transformer	0.884±0.007	0.934±0.009	0.092±0.005	0.851±0.008	0.918±0.010	0.112±0.006	0.836±0.009	0.901±0.010	0.124±0.006	<0.001
Causal Temporal Graph Network	0.902±0.007	0.948±0.008	0.079±0.004	0.871±0.008	0.935±0.009	0.094±0.005	0.859±0.008	0.922±0.009	0.103±0.005	<0.001
DST2former	0.909±0.006	0.952±0.008	0.073±0.004	0.883±0.007	0.941±0.008	0.087±0.004	0.871±0.007	0.930±0.008	0.095±0.005	0.004
TDMGCN	0.913±0.006	0.955±0.007	0.070±0.004	0.887±0.007	0.944±0.008	0.083±0.004	0.876±0.007	0.934±0.008	0.091±0.004	0.003
MGSGDM	0.918±0.005	0.960±0.006	0.067±0.003	0.894±0.006	0.949±0.007	0.078±0.004	0.882±0.006	0.941±0.007	0.086±0.004	0.002
Proposed Method	** 0.927±0.004 **	** 0.966±0.005 **	** 0.061±0.003 **	** 0.904±0.005 **	** 0.957±0.006 **	** 0.072±0.003 **	** 0.893±0.005 **	** 0.948±0.006 **	** 0.081±0.004 **	–

**Table 4:** Results of the ablation study (mean ± standard deviation over five runs).

Variant	MAE	RMSE	MAPE (%)	Accuracy	Precision	Recall	F1	AUC	CDR	*p*-Value
w/o Road Image Features	3.47±0.042	5.59±0.067	11.72±0.154	0.886±0.006	0.872±0.007	0.861±0.008	0.866±0.007	0.931±0.006	0.861±0.008	0.018
w/o GPS Trajectory Features	3.52±0.045	5.67±0.071	11.91±0.161	0.881±0.007	0.868±0.008	0.853±0.009	0.860±0.008	0.927±0.007	0.853±0.009	0.012
w/o Public Transportation Features	3.43±0.041	5.52±0.065	11.58±0.150	0.889±0.006	0.876±0.007	0.864±0.008	0.870±0.007	0.934±0.006	0.864±0.008	0.026
w/o Weather and Event Features	3.61±0.048	5.83±0.075	12.24±0.169	0.873±0.008	0.859±0.009	0.842±0.010	0.850±0.009	0.918±0.008	0.842±0.010	0.006
w/o Reliability-Aware Fusion	3.73±0.052	6.01±0.081	12.68±0.181	0.864±0.009	0.847±0.010	0.834±0.011	0.840±0.010	0.907±0.009	0.834±0.011	0.003
w/o Dynamic Causal Graph Learning	3.89±0.057	6.27±0.087	13.19±0.193	0.852±0.010	0.836±0.011	0.819±0.012	0.827±0.011	0.896±0.010	0.819±0.012	<0.001
w/o Temporal Transformer	3.78±0.054	6.09±0.083	12.84±0.187	0.861±0.009	0.845±0.010	0.831±0.011	0.838±0.010	0.904±0.009	0.831±0.011	0.002
w/o Causal Explanation Constraint	3.58±0.046	5.76±0.073	12.06±0.164	0.878±0.007	0.862±0.008	0.849±0.009	0.855±0.008	0.922±0.007	0.849±0.009	0.009
Fixed Topology Graph Instead of Dynamic Graph	3.96±0.061	6.38±0.091	13.46±0.201	0.846±0.011	0.829±0.012	0.812±0.013	0.820±0.012	0.889±0.011	0.812±0.013	<0.001
Simple Concatenation Instead of Attention Fusion	3.82±0.056	6.16±0.085	13.02±0.190	0.858±0.010	0.841±0.011	0.826±0.012	0.833±0.011	0.901±0.010	0.826±0.012	0.001
Proposed Method	** 3.21±0.031 **	** 5.18±0.058 **	** 10.94±0.112 **	** 0.914±0.005 **	** 0.902±0.006 **	** 0.889±0.007 **	** 0.895±0.006 **	** 0.956±0.005 **	** 0.889±0.007 **	–

## Data Availability

The data presented in this study are available on request from the corresponding author.
